# Post Wire-Bonding Corrosion Prevention Strategies to Mitigate Chloride- and Bromide-Induced Corrosion Failures in Cu- and PCC-Based Wire-Bonded Packages

**DOI:** 10.3390/mi16101155

**Published:** 2025-10-12

**Authors:** Dinesh Kumar Kumaravel, Shinoj Sridharan Nair, Khanh Tuyet Anh Tran, Pavan Ahluwalia, Kevin Antony Jesu Durai, Oliver Chyan

**Affiliations:** 1 Department of Chemistry, University of North Texas, Denton, TX 76201, USA; dineshkumarkumaravel@my.unt.edu (D.K.K.); shinojnair@my.unt.edu (S.S.N.); khanhtran5@my.unt.edu (K.T.A.T.); kevinantony@my.unt.edu (K.A.J.D.); 2 Texas Academy of Mathematics and Science, University of North Texas, Denton, TX 76201, USA; pavanahluwalia@my.unt.edu

**Keywords:** Cu and PCC wire bonding, LQFP and MAPBGA packages, reliability failure, chloride and bromide corrosion, metal-selective passivation, phosphonic acid–aluminum oxide coordination

## Abstract

To ensure the highest safety standards in modern automobiles, the industry is constantly adopting zero-defect frameworks, such as AEC-Q100, which aims for defective-parts-per-billion (DPPB) or grade-0 level reliability standards in automotive integrated-circuit (IC) packages. Most contemporary wire-bonded packages use either pure copper (Cu) or palladium (Pd)-coated copper (PCC) wires bonded to aluminum (Al) bond pads as interconnections. This choice is made due to their lower cost and superior electrical and mechanical performance, compared to traditional gold wire-based devices. However, these Cu–Al wire-bonded interconnections are prone to ion-induced lift-off/open-circuit corrosion failures when exposed to even trace amounts (<20 ppm) of extrinsic and/or intrinsic halide (Cl^−^ and Br^−^) contaminants, decreasing device longevity. This study investigates corrosion failure mechanisms in Cu and PCC wire-based devices by subjecting non-encapsulated devices to a highly accelerated aqueous-immersion screening test containing 100 ppm chloride (Cl^−^), 100 ppm bromide (Br^−^), and a mixed-ion solution (MX: Cl^−^ + Br^−^). The screening results indicate that even control PCC-Al devices with a Pd overlayer can be susceptible to Cl^−^ and Br^−^ induced corrosion, with 21 ± 1.6% lift-off failures in MX-solution. In contrast, applying a novel Cu-selective passivation reduced lift-off to 3.3 ± 0.6% and introducing phosphonic-acid-based inhibitor into the MX solution eliminated lift-off failures, demonstrating markedly improved reliability.

## 1. Introduction

Wire bonding remains one of the most mature and widely deployed interconnect technologies in current IC packages. Its continued prevalence is mainly due to its cost effectiveness and compatibility with high-volume manufacturing. Recent heterogeneous integration schemes even combine wire bonding with 3D die stacking in the same devices to reduce costs and enhance scalability [1].

**Figure 1:**
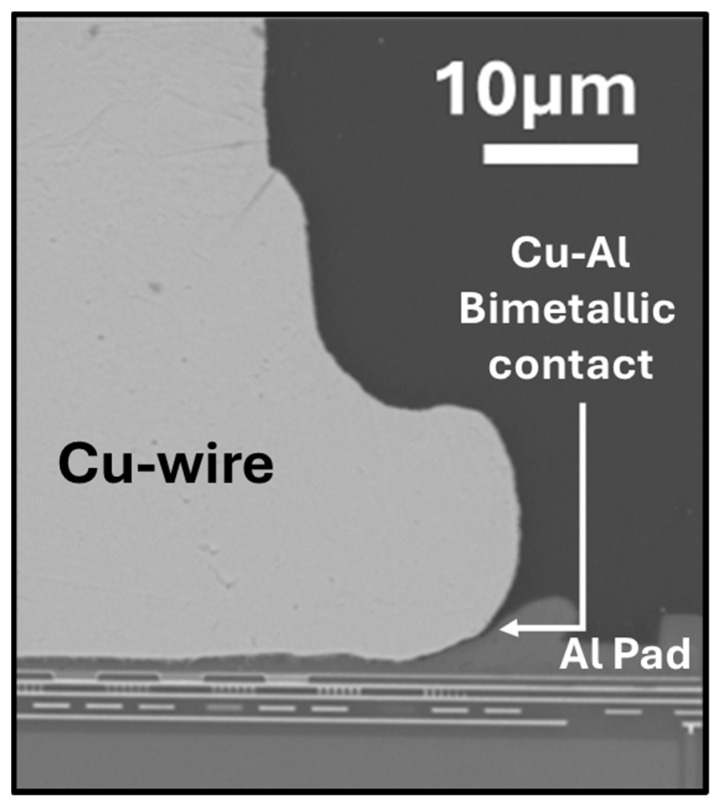
SEM cross-sectional image of a Cu-wire-bonded device, highlighting the Cu–Al bimetallic contact.

Over the past decade, the industry has shifted from gold (Au) and silver (Ag) wires to copper (Cu) wires bonded to aluminum (Al) pads, and it is widely adopted across most applications. Aside from the cost, previous studies indicated that Au–Al has poor reliability at high temperatures, and Ag–Al is susceptible to corrosion and electromigration, making copper a much-preferred choice [2,3,4]. Additionally, Zhong et al. and Zhou et al. have provided in-depth analyses of the advantages and current challenges associated with the use of Cu wires, emphasizing their superior electrical and thermal conductivity, high mechanical strength, slower intermetallic compound (IMC) growth, propensity for corrosion, and increased susceptibility to oxidation [2,5]. Notably, high-temperature annealing studies of Cu–Al wire-bonded devices conducted by C.-P. Liu et al. simulated real-world operating environments and demonstrated the sequential formation of three intermetallic compounds: CuAl_2_, CuAl, and Cu_9_Al_4_ at the Cu/Al interfaces [6]. These phases, along with the parent Cu and Al at the bimetallic interface, exhibit sizable galvanic potential differences. When coupled with the hydrophilic epoxy molding compound (EMC), which shows accelerated water uptake and ionic migration, such galvanic interactions create an environment that is highly conducive to corrosion [7,8,9,10]. This has been one of the significant challenges for automotive wire bond packages, which are exposed to high-humidity and high-temperature operating conditions. Most reliability studies indicate that Cu_9_Al_4_ and CuAl phases are major corrosion sites, and the concentration of Cl^−^ ions and pH values directly affect the corrosion rate [9,10,11,12,13,14]. V. Mathew et al. employed extraction–immersion testing (EIT) as guideline metrology, alongside biased highly accelerated temperature-and-humidity stress (HAST), to assess the effect of halide concentration, solution pH, and applied bias on lift-off or open-circuit failures. Their results correlate with previous findings, indicating that, at pH lower than or equal to 6, halide levels as low as 20 ppm of Br^−^ and/or Cl^−^ are sufficient to trigger lift-off failures. Moreover, the failure rate increases as the device bias is raised, and results from immersion screening are directly correlated with HAST results [15,16]. N. Ross et al. further investigated the corrosion mechanism of (20 ppm) Cl^−^ ion-induced wire bond failures using aqueous immersion screening metrology. Their study highlighted the Cu–Al bimetallic contact between the Cu wire and Al splash, illustrated in Figure 1, as a significant initiating factor for corrosion. Furthermore, the study identified hydrogen evolution as the major cathodic reaction associated with this corrosion [17].

**Figure 2:**
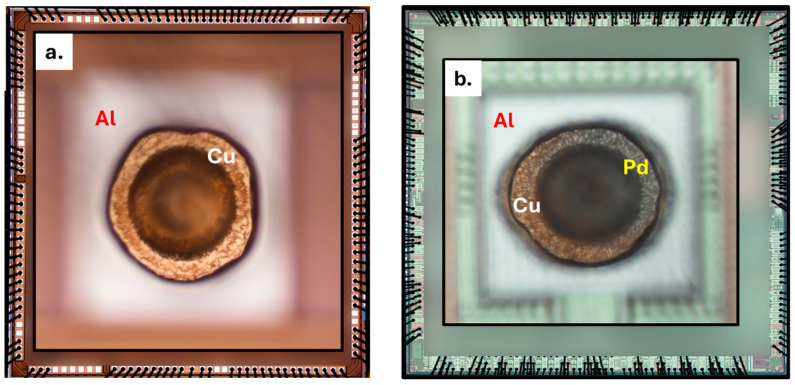
Optical image comparison of (**a**) Cu-Al (134 wire-bonds) and (**b**) PCC-Al (409 wire bonds) wire-bonded devices. Center inset: zoomed-in top-down view of a single ball bond to the bond pad surface.

One obvious way to eliminate these corrosion failures is to eliminate halide ions from the wire-bonding vicinity inside the EMCs [16,17,18]. However, these halide contaminants are difficult to control as they can originate from intrinsic sources (such as EMC, adhesion promoters) and extrinsic sources (such as the fabrication environment and other bill of materials), highlighting the need for alternative strategies [18]. Various researchers have explored metal passivation methods for wires and bond pads, and only the inclusion of Pd in Cu wire has effectively minimized the formation of pure Cu–Al IMCs at the same time without deforming the free air ball (FAB) during wire bonding; this technique has therefore been widely adopted for various applications [2,19]. Lim et al. and Qin et al. report on Pd inclusion in copper wires, detailing the electrochemical and metallurgical aspects of reliability improvement in ionic environments [20,21]. Their results highlight that the Pd inclusion mainly forms Pd-mixed Cu–Al IMCs and reduces the formation of Cu-rich IMC, thereby reducing the corrosion sites. However, during manufacturing, Pd coverage can be difficult to control due to differing Cu and Pd solidification temperatures, potentially exposing some copper areas (as shown in Figure 2b) and reducing reliability [22]. As an alternative approach, C.-P. Liu et al. tested the effect of incorporating corrosion inhibitors (CI) and flame retardants (FR) within EMCs to evaluate their effectiveness in mitigating Cu–Al device failures under HAST conditions [9]. Their results showed that including CI and FR significantly increases the mean time to failure (MTTF) compared to unmodified control EMCs. However, even in the presence of CIs and FRs, an increase in chloride ion (Cl^−^) concentration still led to higher failure rates. A recent study evaluated epoxy resins, bismaleimides (BMI), and Parylene-based polymers as coatings applied after wire bonding for PCC–Al devices. They aimed to eliminate halide ions from the immediate vicinity (<100 µm) and assessed their effectiveness in reducing Cl^−^-induced corrosion failures as well as their compatibility with manufacturing [18]. The results showed that only epoxy resins are suitable for manufacturing processes, while the other two materials failed thermal reliability tests. Further research is needed to identify and study new materials that could serve as post-wire-bonding coatings to eliminate halide-induced corrosion failures in Cu–Al wire-bonded devices.

This study utilizes accelerated aqueous immersion stress tests to systematically compare the corrosion trends of Cu- and PCC-based wire-bonded devices, investigating their lift-off failure mechanisms in 100 ppm Cl^−^, Br^−^, and mixed-ionic (100 ppm Cl^−^ + 100 ppm Br^−^) environments. This method allows for rapid and highly aggressive corrosion quantification since ionic diffusivity is higher in aqueous solutions (on the order of 10^4^ cm^2^/s greater) than typical EMCs, and higher ionic concentrations were utilized, compared to the levels of less than 20 ppm typically present in EMCs [23,24,25]. Furthermore, two complementary novel passivation approaches are introduced: (i) a copper-selective overcoat applied after wire bonding, and (ii) an organic phosphonic-acid-based inhibitor in screening solutions. The copper-selective passivation with precise thickness control can conformally coat only the copper-exposed ball area. This may eliminate the cathodic reactions on Cu without affecting other materials in an IC package, making it compatible with manufacturing processes and suitable for integration into real-world microelectronic devices. Phosphonic-acid-based organic molecules are known to form complexes with metal oxides, offering high tunability and surface modification capabilities [26]. Some recent reports have explored the utilization of phosphonate or phosphonic-acid-based complexes for preventing corrosion on Al and its alloys [27,28,29]. Nevertheless, the effectiveness of organic phosphonic acid complexes in suppressing corrosion induced by Cl^−^ and Br^−^ on aluminum bond pads remains unexplored in wire-bonded IC packages. This study presents a novel phosphonic-acid-based passivation treatment for Al bond pads in both Cu–Al and PCC-Al wire-bond assemblies, delivering robust inhibition of bimetallic corrosion at exposed Cu–Al interfaces and markedly enhancing package reliability by preventing lift-off failures and ensuring functional safety in automotive ICs.

## 2. Materials and Methods

### 2.1. Wirebond Devices

This study utilizes two types of wire-bonded devices, as shown in Figure 2. The first type consists of 5 mm × 4.6 mm functional dies, which are surface-mounted on Cu-based lead frames from low-profile quad flat packages (LQFP). These devices featured Cu wires (approximately 25 µm in diameter) that were thermosonically wire-bonded to Al pads measuring 70 µm × 78 µm on the dies. Throughout this article, these devices will be referred to as “Cu-Al Devices.” Similarly, the second type of device consists of dies measuring approximately 5.5 mm × 5.5 mm, and they were surface mounted onto the lead frames from a mold array process ball grid array (MAPBGA) packages. These devices used PCC wires of approximately 20 µm diameter for ball bonding and will be referred to as “PCC-Al devices”. All the devices were plasma cleaned to remove any particle contamination with a Harrick Plasma etcher using 30 W power and 5 SCCM of 95% Ar-H_2_ gas mixture for 5 min before being subjected to any treatment.

### 2.2. Accelerated Immersion Screening Metrology

**Figure 3:**
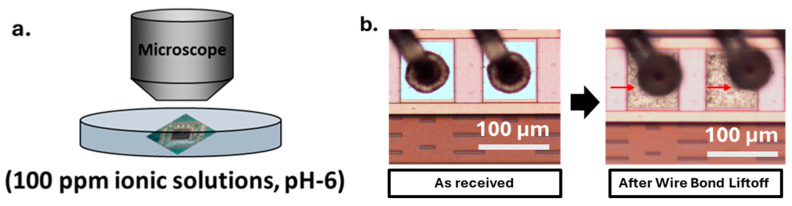
(**a**) Schematics of the corrosion screening metrology and (**b**) microscopic images of wire bond lift-off considered as failure defects for assessing corrosion and statistical analysis.

The corrosion screening solutions containing 100 ppm chloride (Cl^−^), 100 ppm bromide (Br^−^), and mixed ions (MX: 100 ppm Cl^−^ and 100 ppm Br^−^ ions) were prepared by dissolving ACS-grade sodium chloride (EMDMillipore Corporation, Billerica, MA, USA) and potassium bromide (Fisher Scientific, Fair Lawn, NJ, USA) in ultrapure water (UPW) obtained from a EQ-7008 (Millipore Sigma, Burlington, MA, USA) water system with a resistivity of 18.2 MΩ·cm. The screening solutions were adjusted to pH 6 using ACS-grade ammonium hydroxide (Fisher Chemical, Fair Lawn, NJ, USA) and sulfuric acid (Fisher Scientific, Fair Lawn, NJ, USA) solutions to emulate the typical pH reported in the EMCs [16]. The corrosion process was monitored in real-time using a Nikon Eclipse LV 150N microscope (New York, NY, USA), and the time-dependent images were captured using a DS-Ri2 camera (New York, NY, USA). For further corrosion quantification and comparison, the number of lifted wire bonds, similar to those in Figure 3b, was counted and used to calculate the wire bond lift-off percentage and then plotted in Origin software (Version 2025b).

### 2.3. Passivation Approach and Quantification Metrologies

As described earlier, two distinct targeted passivation approaches were explored in this study. First, a copper selective passivation film was applied to Cu–Al and PCC–Al wire-bonded devices. Reflection–absorption infrared spectroscopy (RAIRS) was conducted using a Thermo-Nicolet iS50 (Dallas, TX, USA) spectrometer for chemical quantification and tuning passivation thickness. The exact method of calibration for this metrology is discussed in our previous report [30]. Based on the calibration, the passivation thickness of 2–3 microns was coated on Cu-Al and PCC-Al devices. The passivation chemistry and the processing steps are detailed in our previous work and patent application [31]. Typically, the passivation process may include a plasma cleaning, a liquid or gas phase coating process, and post-coating rinsing with UPW to remove any physisorption. After the passivation process, contact angle measurements on passivated Cu lead frames (no. of devices = 3) were performed using a YSC M400 power scope (Fremount, CA, USA) by making 10 µL UPW droplets, and the images were analyzed using ImageJ software (version 1.54g).

The second approach introduced Nitrilotris (methylene) phosphonic acid (NTMP) to the corrosion screening solutions. A 50% NTMP (Sigma-Aldrich, St. Louis, MO, USA) solution from Sigma-Aldrich was used to make 0.5% NTMP-contaminated ionic (100 ppm Cl^−^, 100 ppm Br^−^, 100 ppm Cl^−^ + 100 ppm Br^−^) screening solutions and adjusted to pH 6. The Cu–Al and PCC–Al devices were directly immersed in these screening solutions and observed under the microscope to study the corrosion inhibition effectiveness.

### 2.4. SEM, GI-XRD, and XPS Analysis

The JEOL IT 200-Scanning Electron Microscope (SEM) was utilized to image wire bond devices at various stages of our coating and testing processes. The SEM was operated at 5 kV with the standard current value of 50 mA, and both the secondary electron detector (SED) and the backscattered electron detector in composition mode (BED-C) were utilized for imaging purposes. Energy Dispersive X-ray spectroscopy (EDX) analysis was conducted using the same microscope, utilizing T4 resolution and a live scan of 120 s in circular and polygon modes. The EDX-map analysis was performed on non-wire-bonded Al bond pads at 5 kV with a resolution of 512 × 384 pixels and a dwell time of 0.2 ms for 30 min. The grazing incidence X-ray diffraction patterns (GI-XRD) were obtained using a Rigaku Smart lab diffractometer (Woodlands, TX, USA) with the incidence parallel beam angle of 0.5°, and the data were plotted using Origin software (Version 2025b). X-ray Photoemission Spectroscopy (XPS) was performed with a PHI 5000 Versaprobe Scanning XPS microprobe, utilizing an Al-Kα source with an anode voltage of 11 kV and pass energy of 29.35 eV, to analyze the chemical–mechanical planarized (CMP) Al-wafer (0.5% Cu) and Cu-wafer samples. For effective charge calibration, the Al-2p metallic peak was calibrated to 72.9 eV for Al wafers, and the Cu-2p_3/2_ metallic peak was calibrated to 932.7 eV for Cu wafers. The post-processing of XP spectra was performed manually using CasaXPS (Version 2.3.26PR1.0), and the curve fitting was performed using mixed Gaussian–Laurensian [GL(30)] peak profiles, and the full width half-maximum (FWHM) was constrained to be less than 3 eV [32].

### 2.5. Electrochemical Analysis

Potentiodynamic polarization measurements were performed on the CMP polished Al wafers (0.5% Cu) using a CHI-760D potentiostat (Austin, TX, USA). These experiments used ionic screening solutions as electrolytes, platinum (Pt) foil as a counter electrode, and silver/silver chloride (Ag/AgCl) as a reference electrode. The working electrode was prepared by masking the Al-wafer samples using a polyimide tape, exposing only a 1 cm^2^ active area. Each sample was immersed in the solution for at least 15 min before conducting open-circuit potential (OCP) measurements. Polarization curves were obtained within a range of ±100 mV at a scan rate of 1 mV/s relative to the OCP measurements. The corrosion potential, E_corr_, was determined based on the intersection of the anodic and cathodic polarization curves. As shown in Figure S5, the intersection point of slopes drawn from the cathodic and anodic branches to the E_corr_ line was taken for corrosion current, I_corr_.

## 3. Results and Discussion

### 3.1. Accelerated Corrosion Screening Analysis

**Figure 4:**
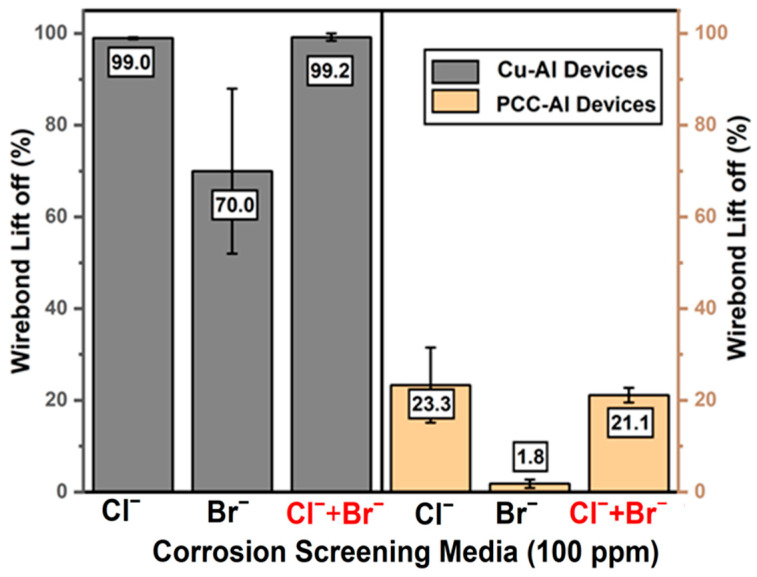
Accelerated screening results on Cu–Al and PCC–Al devices showing extensive corrosion in ionic screening solutions after 2 h, based on wire-bond lift-off counts (no. of devices tested per condition = 3).

We first tested the corrosion performance of control Cu and PCC wires by subjecting Cu–Al and PCC–Al devices to highly accelerated immersion testing in 100 ppm ionic solutions (Cl^−^, Br^−^, Cl^−^ + Br^−^) for 2 h. The Cu–Al devices with pure Cu wire underwent severe corrosion, with 99 *±* 0.2% wire-bond lift-off in Cl^-^ media and 70 *±* 18% wire-bond lift-off in Br^−^ based ionic screening. Although the industrial-standard PCC-Al devices performed better in the Br^−^ solution (1.8 *±* 0.9%), they still showed 23 ± 8.2% wire-bond lift-off in the Cl^−^ solution, indicating severe corrosion vulnerability. Notably, chloride ions predominantly influenced the corrosion behavior in the MX solution, yielding results identical to those observed in the pure Cl^−^ solution (Figure 4).

**Figure 5:**
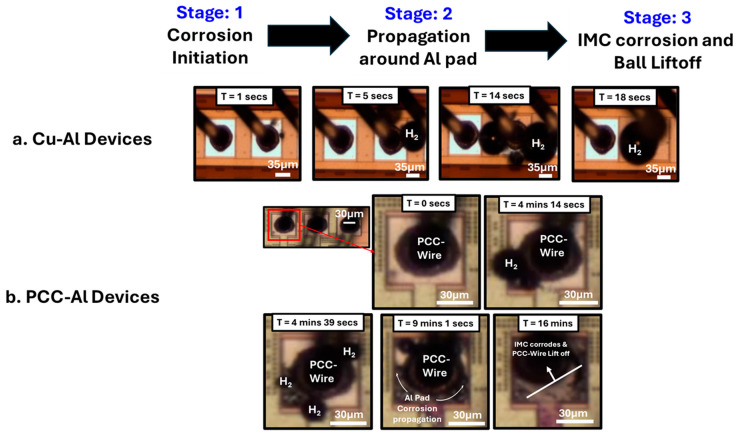
Real-time corrosion mechanism of Cu–Al device in mixed ion screening solution (100 ppm Cl^−^ and 100 ppm Br^−^).

Figure 5 demonstrates the time-dependent corrosion progression in wire bond devices observed under mixed ionic screening conditions. The corrosion mechanism for both Cu–Al and PCC–Al followed a three-stage process:

1. Corrosion initiation is characterized by bubble formation at the Cu–Al interface.
2. Propagation of Al pad corrosion, leading to dendrite and mud crack formation in the aluminum pads.
3. Corrosion of the underlying IMCs ultimately results in the ball lift-off.

This corrosion progression aligns with previously reported Cl^−^-based mechanistic studies by N. Ross et al., in which they demonstrated that, in addition to the IMCs, the Cu–Al (Cu to Al splash) bimetallic contact is another critical factor for corrosion failures, and the bubbles observed were the result of cathodic hydrogen evolution [17]. In our case, the corrosion rate is faster as we tested under 100 ppm Cl^−^ and Br^−^ instead of 20 ppm as mentioned elsewhere [17]. In PCC–Al devices, the time taken for the final IMC corrosion leading to lift-off varied from a few minutes to more than 30 min. This slower corrosion rate and the high variation in the lift-off percentage can be attributed to the difference in the distribution of Pd in the Cu–Al IMCs. The corrosion mechanism for the Br^−^ solution also followed a similar trend for both Cu–Al and PCC–Al devices, with a reduced lift-off percentage (Figure 4) being the only difference.

### 3.2. Optical and SEM Comparison on Passivated Wirebond Devices

**Figure 6:**
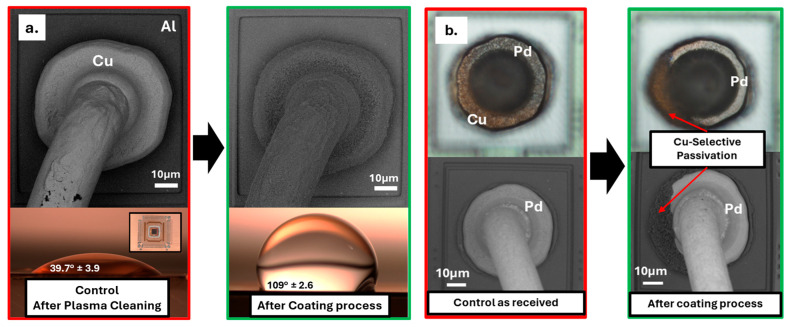
Optical and back-scattered SEM comparison of control and passivated (**a**) Cu–Al and (**b**) PCC–Al wire-bonded devices, demonstrating a conformal passivation. (No: of leadframes tested for contact angle: 3).

The optical and SEM-EDX analysis of control PCC–Al devices revealed the presence of copper-rich and palladium-rich regions, as shown in Figure 6b and Figure S1. We hypothesize that the Cu-rich regions in PCC-Al devices, which retain the Cu–Al bimetallic contact (Figure 1), are responsible for similar corrosion behavior. Following SEM-EDX analysis, the wire-bonded devices were subjected to the Cu-selective passivation process. After the coating process, SEM-backscattered images (Figure 6) of the Cu–Al and PCC–Al devices displayed a passivation layer formation that was conformal only to the copper exposed area across all wire bonds. The subsequent contact angle measurements from the lead frames of Cu-Al devices consistently indicated increased hydrophobicity, indicating the presence of uniform passivation across the entire Cu lead frames (Figure 6a). These devices will be referred to as “passivated Cu-Al devices” and “passivated PCC-Al devices.” In our previous work, passivated PCC–Al devices were subjected to film stability and thermal reliability tests including temperature cycling (TC: −55 °C to 150 °C), temperature humidity bias (THB: 85 °C/85% RH), and High-Temperature Storage Life (HTSL: 175 °C) and passed electrical and delamination failure analyses [22].

### 3.3. Accelerated Corrosion Screening Results on Passivated Devices

**Figure 7:**
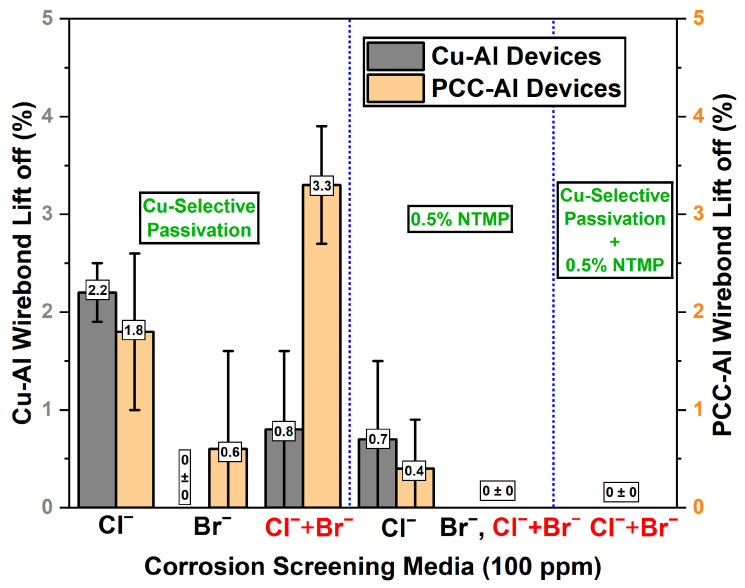
Accelerated screening results on Cu–Al and PCC–Al devices with copper selective passivation, 0.5% NTMP in ionic screening solutions, and the combined approach after 2 h. (No. of devices tested per condition = 3).

**Figure 8:**
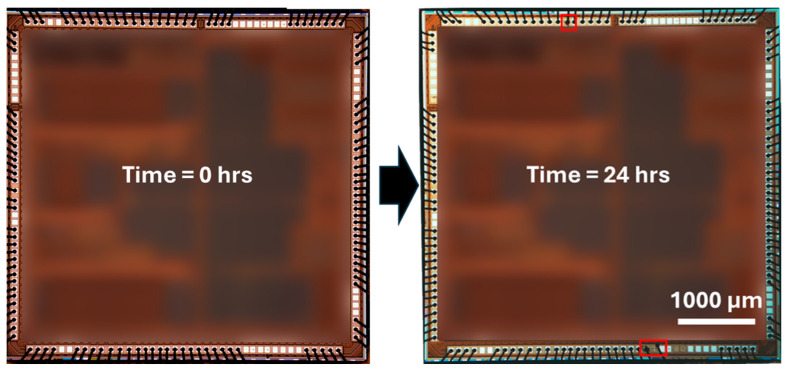
Optical image of Cu–Al device before and after 24-h immersion in 0.5% NTMP contaminated Cl^-^ screening with annotated (red box) ball lift-off areas.

As shown in Figure 7, the passivated Cu–Al and PCC–Al devices showed significant improvements with at least ~45× and ~7× reductions in ball lift-off percentage across all the ionic solutions compared to the control Cu–Al and PCC–Al devices (Figure 4). These results highlight that reducing cathodic half-cell reactivity by passivating the exposed Cu area (Figure 6) decreases the lift-off failures in the wire-bonded devices. Furthermore, Figure 7 demonstrates the effect of 0.5% NTMP inclusion in ionic solutions, which exhibited excellent corrosion inhibition, achieving less than 1.5% lift-off in most ionic screening conditions. Even after 24 h of immersion in a 0.5% NTMP-added 100 ppm Cl^−^ solution (Figure 8), the Cu–Al devices showed superior corrosion performance, with less than 3% ball lift-off. Furthermore, applying the combined approach, Cu-selective passivation, along with 0.5% NTMP inclusion in the screening solution, achieved 0% ball lift-off consistently across both types of devices, indicating excellent reliability.

### 3.4. Post-Screening SEM Analysis

**Figure 9:**
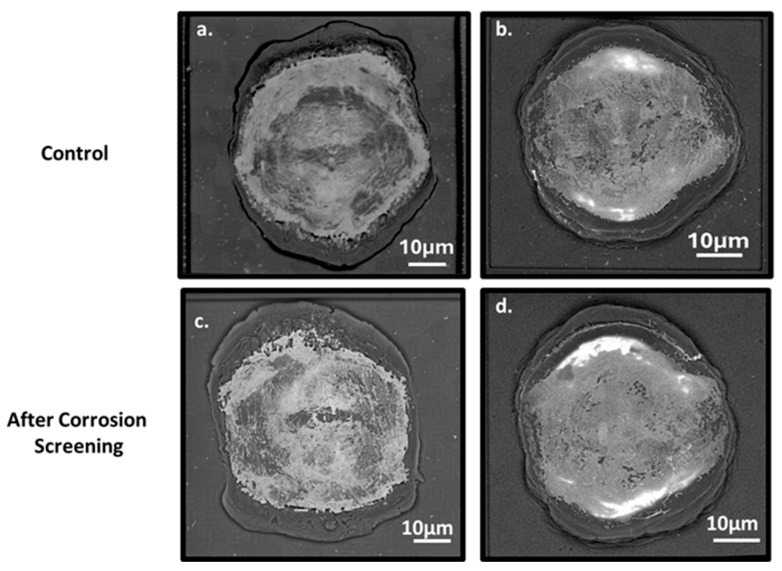
Back-scattered SEM images of control and corrosion-screened Cu–Al (**a**,**c**) and PCC–Al (**b**,**d**) devices, indicating similar IMC presence.

After two hours of immersion screening, the Cu–Al and PCC–Al devices were further examined for microscopic IMC degradation by etching away the copper wires [33]. This method is widely used to determine cracks and corrosion degradation in the IMC region and can be used to measure the contact resistance variation and the bond strength in the wire-bonded interconnects [34]. As illustrated in the SEM backscattered images (Figure 9), the bright center region corresponds to the IMC present between the Cu wire and the Al Pad. The results reveal that the IMCs in the non-corroded Al pads, following corrosion screening, were similar to those in the control Al pads, showing no visible signs of IMC degradation. This suggests that preventing corrosion initiation at the Cu–Al bimetallic interface can effectively reduce halide-ion-induced IMC degradation, with no impact on contact resistance. Additionally, SEM images of Cu–Al devices (Figure S2) after 24 h screening did not show any Al pad structure modification. However, the EDX area analysis on non-wire-bonded Al pads detected a minor presence of phosphorus in Al pad surfaces (Figure S2). At the same time, EDX-mapping did not show a significant difference between control and NTMP-treated samples (Figures S3 and S4). This may be due to the higher penetration depth of the EDX, and the surface modification on Al might be relatively thin.

### 3.5. NTMP Interactions with Al and Cu Surface

**Figure 10:**
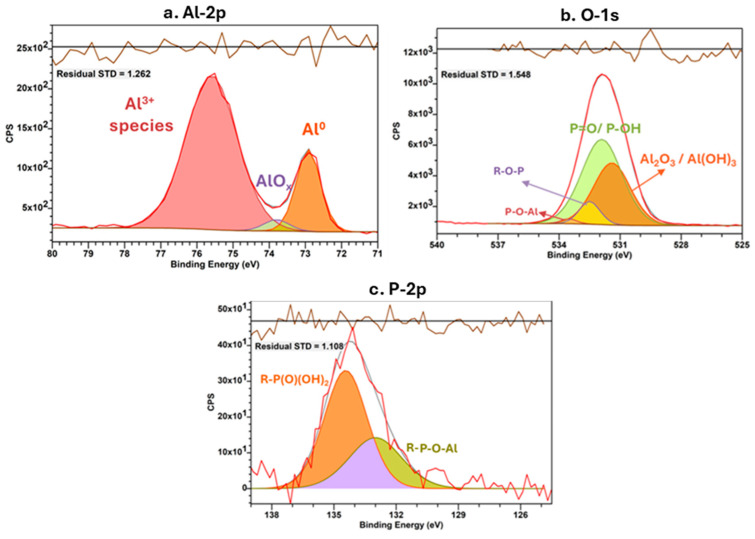
(**a**–**c**) XP spectra of Al wafers immersed in NTMP-contaminated Cl^−^ solution show passivation formation on the Al surface. (Colored regions represent the deconvoluted components as shown in the Table 1).

To investigate NTMP-based surface modification on Al pads, Al (0.5% Cu) wafers, which have a similar chemical composition to the Al bond pads, were immersed in NTMP-contaminated 100 ppm Cl^−^ solutions for 24 h. The treated Al wafers were then rinsed with UPW, and XPS analyses were performed (Figure 10). The aluminum 2p XP spectrum, in Figure 10a, can be resolved into three peaks at 72.9 eV, 73.8 eV, and 75.6 eV. The lower-energy peak is associated with metallic aluminum, which has unresolved 2p_3_/_2_ and 2p_1_/_2_ orbital coupling. The peak at 73.8 eV can correspond to AlO_x_ in the Al_2_O_3_ network [35]. The higher-energy component is likely due to overlapping contributions from aluminum oxide, aluminum hydroxide, and aluminum phosphonates [29,35,36,37,38,39,40]. Previous XPS studies examining the Al valence band region after phosphonic acid treatment also indicated challenges in distinguishing contributions due to the dominance of amorphous Al_2_O_3_ at the surface [28]. Additionally, the peak separation between Al (0) and Al_2_O_3_ is slightly different (2.7 eV) from the value of 2.9 eV reported in the literature, which may be due to the contributions from hydroxides and phosphates [39]. Xia et al. demonstrated through experimental and computational studies that aluminum substrates with Lewis acidic oxides and/or hydroxides can interact with the P-O group from phosphonic acids. This interaction renders the phosphorus (P) more electrophilic and promotes heterocondensation, ultimately forming stable P-O-Al groups on the aluminum surface [29].

The broad oxygen 1s X-ray Photoelectron Spectroscopy (XPS) spectrum, summarized in Table 1, shows contributions from several groups, including Al_2_O_3_/Al (OH)_3_, P = O/P-O-H, P-O-R, P-O-Al coordination groups [36]. The phosphorus 2p XPS peak can be deconvoluted into two distinct components. The higher-energy component at 134.4 eV is associated with a free, non-bonded phosphonic acid group, while the lower energy component at 133 eV aligns well with reported values for phosphorus anchoring groups found in typical metal oxide–phosphonate coordination [36,41,42]. Additionally, the peak area ratio between free phosphonic acid and metal phosphonates is 1.88:1, indicating that NTMP forms a mixture of monodentate and bidentate coordination modes with the aluminum surface, with monodentate binding being the more prevalent. These findings confirm the stable formation of chemical bonds between NTMP and the aluminum oxide on the Al wafers and bond pads. Furthermore, these chemically bonded complexes can serve as a barrier on the aluminum oxide surface, protecting it from Cl^−^ and/or Br^−^ attack. Additionally, Tafel analysis (Figure S5) indicates a notable increase in corrosion potential and a decrease in corrosion current density for the Al wafers immersed in NTMP-contaminated solution compared to those in a control solution containing 100 ppm Cl^−^. This behavior is attributed to the passivation effect.

**Figure 11:**
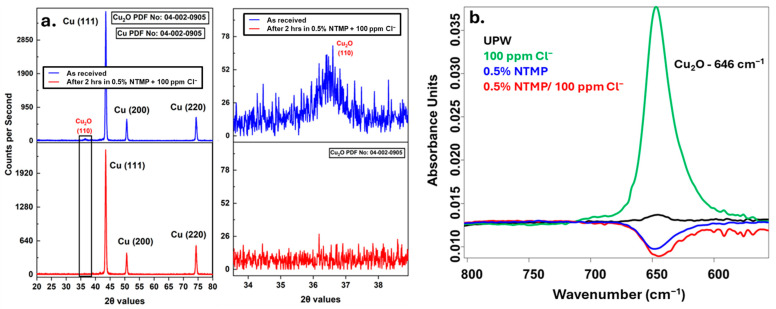
(**a**) Grazing incident X-ray diffraction and (**b**) RAIRS analysis of Cu wafers in various solutions.

In contrast to the Al wafers, the metal phosphonate peak was not observed in the P-2p spectra of NTMP-treated Cu-wafers (Figure S6). To further investigate the interaction between copper oxide and the NTMP screening solutions, we utilized grazing incidence X-ray diffraction (GI-XRD) and reflection–absorption infrared spectroscopy (RAIRS). As illustrated in Figure 11, the GI-XRD results confirm the presence of Cu_2_O on the surface of as-received copper wafers. Upon immersing the wafers in NTMP screening solutions for 2 h, the GI-XRD indicated the dissolution of the copper oxide layer from the Cu wafer. Moreover, the RAIRS (Figure 11b) comparison between control 100 ppm Cl^-^ and NTMP contaminated solutions showed that the copper oxide stretching peak decreases only when NTMP was present in the solution [30]. These findings show that copper oxides form easily dissolvable complexes with NTMP in aqueous solutions, while the Al-oxides form a stable complex. Similarly, Kozlica et al. reported comparable behavior in their corrosion studies, where octyl phosphonic acid served as an inhibitor for aluminum but not for copper [36].

## 4. Conclusions

This study systematically compared the corrosion tendency, failure rate, and underlying corrosion mechanisms of Cu and PCC wire-bond devices in the presence of two ubiquitous ionic contaminants (Cl^−^ and Br^−^) in an IC manufacturing process. Both ions showed similar three-stage corrosion progression under aqueous immersion screening conditions; however, the Br^−^ ions exhibited a slower failure rate. Under highly accelerated immersion screening tests, the PCC–Al devices performed better than Cu–Al devices but still suffered severe corrosion in the presence of Cl^−^ ions, with 23 ± 8.2% lift-off failures within 2 h of immersion. This behavior was attributed to the presence of exposed copper areas in the thermosonic ball region.

In the second part of this study, we explored copper-selective passivation and NTMP-based aluminum passivation strategies to prevent wire bond failures induced by Cl^−^ and Br^−^. The backscattered SEM and contact angle analysis confirmed the conformal Cu-selective film formation on Cu exposed areas in Cu-Al and PCC-Al devices. Even in extreme MX screening conditions, copper selective passivation demonstrated 97 ± 0.2% and 96 ± 0.6% corrosion protection for Cu–Al and PCC–Al devices, respectively. Similarly, including 0.5% NTMP in the screening solutions also improved corrosion protection, with only 0–1% lift-off across all the solutions. XPS and potentiodynamic polarization confirm that NTMP forms a stable phosphonate–aluminum-oxide coordination layer on the Al bond pads, while GI-XRD and IRRAS reveal that Cu-oxide–phosphonate complexes remain unstable in aqueous media. These results collectively show that NTMP eliminates corrosion by suppressing Cl^−^ and Br^−^ attack towards the Al pad, thereby improving the reliability of wire-bonded devices.

## Figures and Tables

**Table 1:** XPS curve fitting results of Al-2p, O-1s and P-2p after NTMP immersion.

Element	Components	Binding Energy	Atom%	References
Al-2p	Al (0) metal	72.9	19.6	[35,36,37,38,39]
	AlO_x_	73.8	2.9	
	Al^3+^ species	75.6	77.5	
O-1s	Al_2_O_3_/Al (OH)_3_	531.4	36.6	[36]
	P = O/P-OH	531.9	54.61	
	P-O-R	532.5	7.61	
	P-O-Al	533.5	1.23	
P-2p	R-P(O)(OH)_2_	134.4	65.3	[36,41,42]
	R-P-O-Al	133.0	34.7	

## Data Availability

Email the corresponding author for any data availability.

## Supplementary Materials

The following supporting information can be downloaded at: https://www.mdpi.com/article/10.3390/mi16101155/s1, Figure S1. SEM-EDX Analysis of the Control non-passivated PCC–Al devices; Figure S2. SEM-EDX Analysis of Cu–Al devices after 24 h of immersion in 0.5% NTMP contaminated 100 ppm Cl^−^ solution indicated no structural change to the Al bond pad, and EDX-analysis detected a minor presence of a phosphorus signal; Figure S3. SEM-EDX Map Analysis of Cu–Al devices after 24 h of immersion in 0.5% NTMP contaminated 100 ppm Cl^−^ solution indicated a non-quantifiable presence of a phosphorus signal; Figure S4. SEM-EDX Map Analysis on PCC–Al devices after 24 h of immersion in 0.5% NTMP contaminated 100 ppm Cl^−^ solution indicated a non-quantifiable presence of a phosphorus signal; Figure S5. Potentiodynamic polarization plots confirming the reduction in Al wafer reactivity in the presence of NTMP; Figure S6. XP spectra of P-2p on Cu wafers in NTMP solutions indicate an absence of P-O-Cu peak at 133 eV after water rinsing.

## Abbreviations

The following abbreviations are used in this manuscript:

PCC	Palladium-coated copper
IC	Integrated circuit
LQFP	Low-profile quad flat packages
MAPBGA	Mold array process ball grid array
EMC	Epoxy molding compounds
NTMP	Nitrilotris (methylene)phosphonic acid
IMC	Inter-metallic compounds
MX	Mixed-ion screening solution
