# Supplementary material for: Post Wire-Bonding Corrosion Prevention Strategies to Mitigate Chloride- and Bromide-Induced Corrosion Failures in Cu- and PCC-Based Wire-Bonded Packages
Source: Micromachines (Basel). 2025 Oct 12;16(10):1155. doi: 10.3390/mi16101155 (PMC12565955; doi:10.3390/mi16101155)
Supplement: Supplementary file 1 [file micromachines-16-01155-s001.zip › micromachines-3543753-supplementary.pptx]

## Slide 1
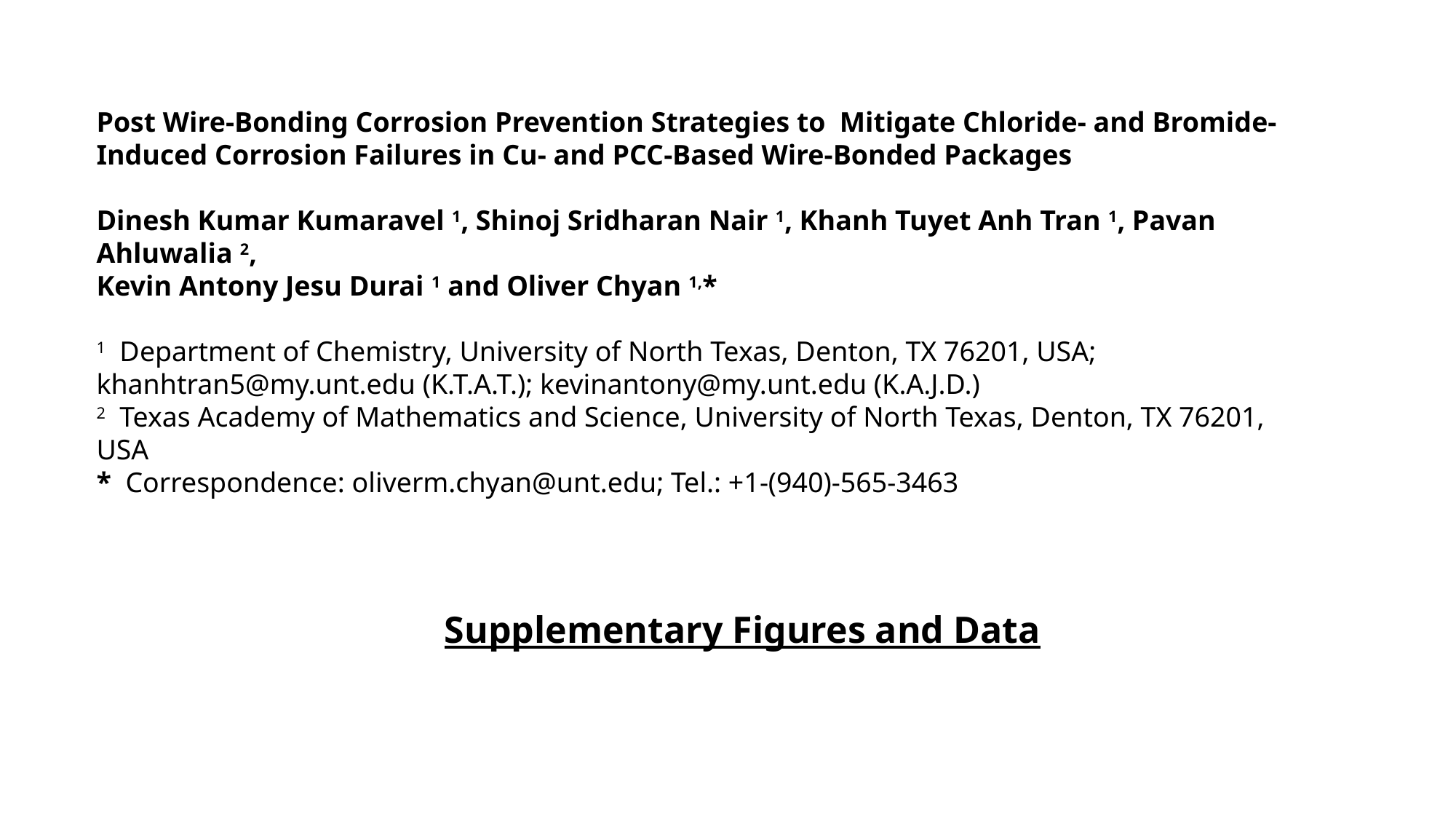

Post Wire-Bonding Corrosion Prevention Strategies to Mitigate Chloride- and Bromide-Induced Corrosion Failures in Cu- and PCC-Based Wire-Bonded Packages
Dinesh Kumar Kumaravel 1, Shinoj Sridharan Nair 1, Khanh Tuyet Anh Tran 1, Pavan Ahluwalia 2,
Kevin Antony Jesu Durai 1 and Oliver Chyan 1,*
1 Department of Chemistry, University of North Texas, Denton, TX 76201, USA;
khanhtran5@my.unt.edu (K.T.A.T.); kevinantony@my.unt.edu (K.A.J.D.)
2 Texas Academy of Mathematics and Science, University of North Texas, Denton, TX 76201, USA
* Correspondence: oliverm.chyan@unt.edu; Tel.: +1-(940)-565-3463
Supplementary Figures and Data

## Slide 2
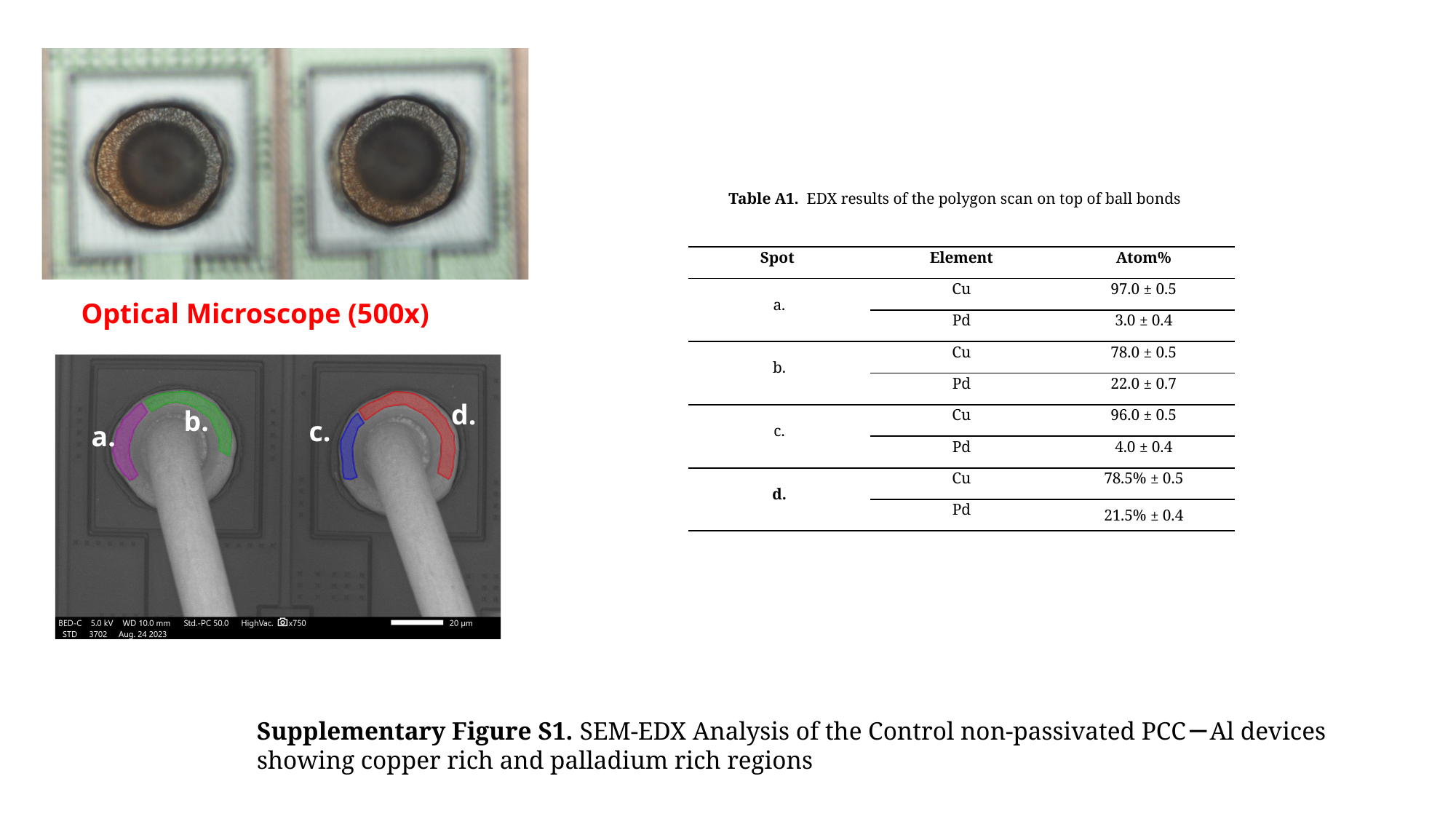

Table A1. EDX results of the polygon scan on top of ball bonds
| Spot | Element | Atom% |
| --- | --- | --- |
| a. | Cu | 97.0 ± 0.5 |
| | Pd | 3.0 ± 0.4 |
| b. | Cu | 78.0 ± 0.5 |
| | Pd | 22.0 ± 0.7 |
| c. | Cu | 96.0 ± 0.5 |
| | Pd | 4.0 ± 0.4 |
| d. | Cu | 78.5% ± 0.5 |
| | Pd | 21.5% ± 0.4 |
Optical Microscope (500x)
d.
b.
c.
a.
Supplementary Figure S1. SEM-EDX Analysis of the Control non-passivated PCC−Al devices showing copper rich and palladium rich regions

## Slide 3
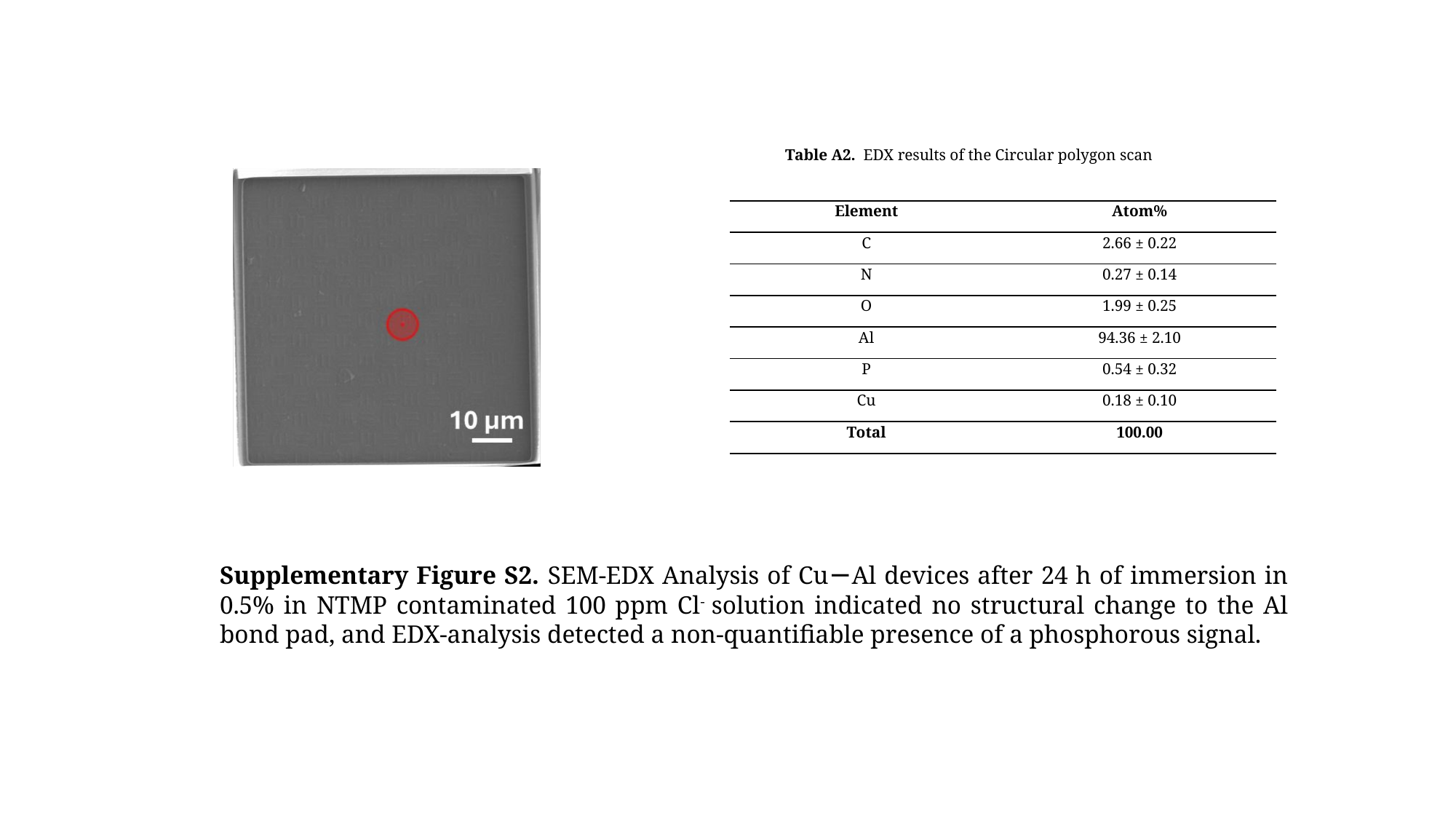

Table A2. EDX results of the Circular polygon scan
| Element | Atom% |
| --- | --- |
| C | 2.66 ± 0.22 |
| N | 0.27 ± 0.14 |
| O | 1.99 ± 0.25 |
| Al | 94.36 ± 2.10 |
| P | 0.54 ± 0.32 |
| Cu | 0.18 ± 0.10 |
| Total | 100.00 |
Supplementary Figure S2. SEM-EDX Analysis of Cu−Al devices after 24 h of immersion in 0.5% in NTMP contaminated 100 ppm Cl- solution indicated no structural change to the Al bond pad, and EDX-analysis detected a non-quantifiable presence of a phosphorous signal.

## Slide 4
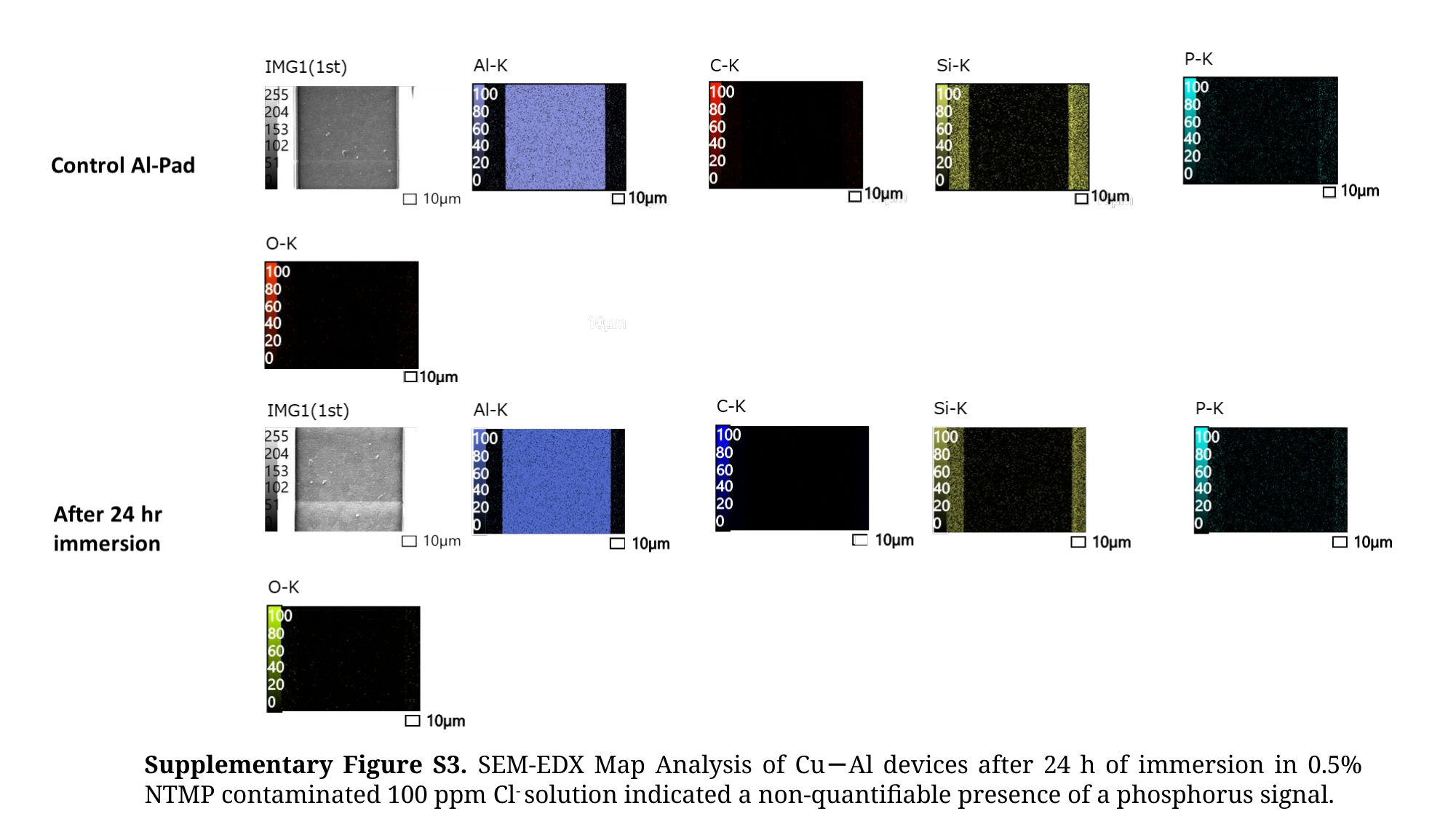

Supplementary Figure S3. SEM-EDX Map Analysis of Cu−Al devices after 24 h of immersion in 0.5% NTMP contaminated 100 ppm Cl- solution indicated a non-quantifiable presence of a phosphorus signal.

## Slide 5
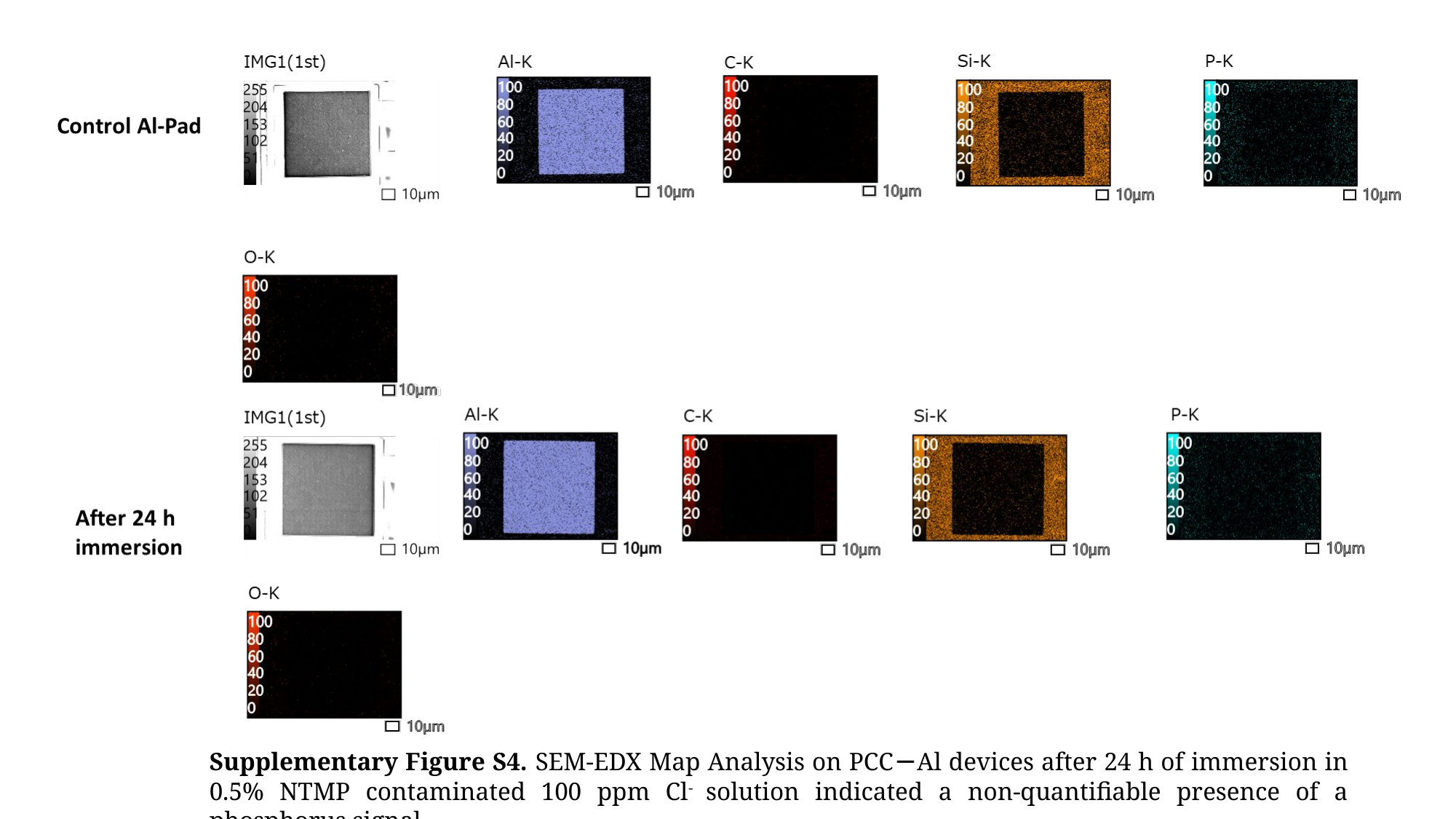

Supplementary Figure S4. SEM-EDX Map Analysis on PCC−Al devices after 24 h of immersion in 0.5% NTMP contaminated 100 ppm Cl- solution indicated a non-quantifiable presence of a phosphorus signal.

## Slide 6
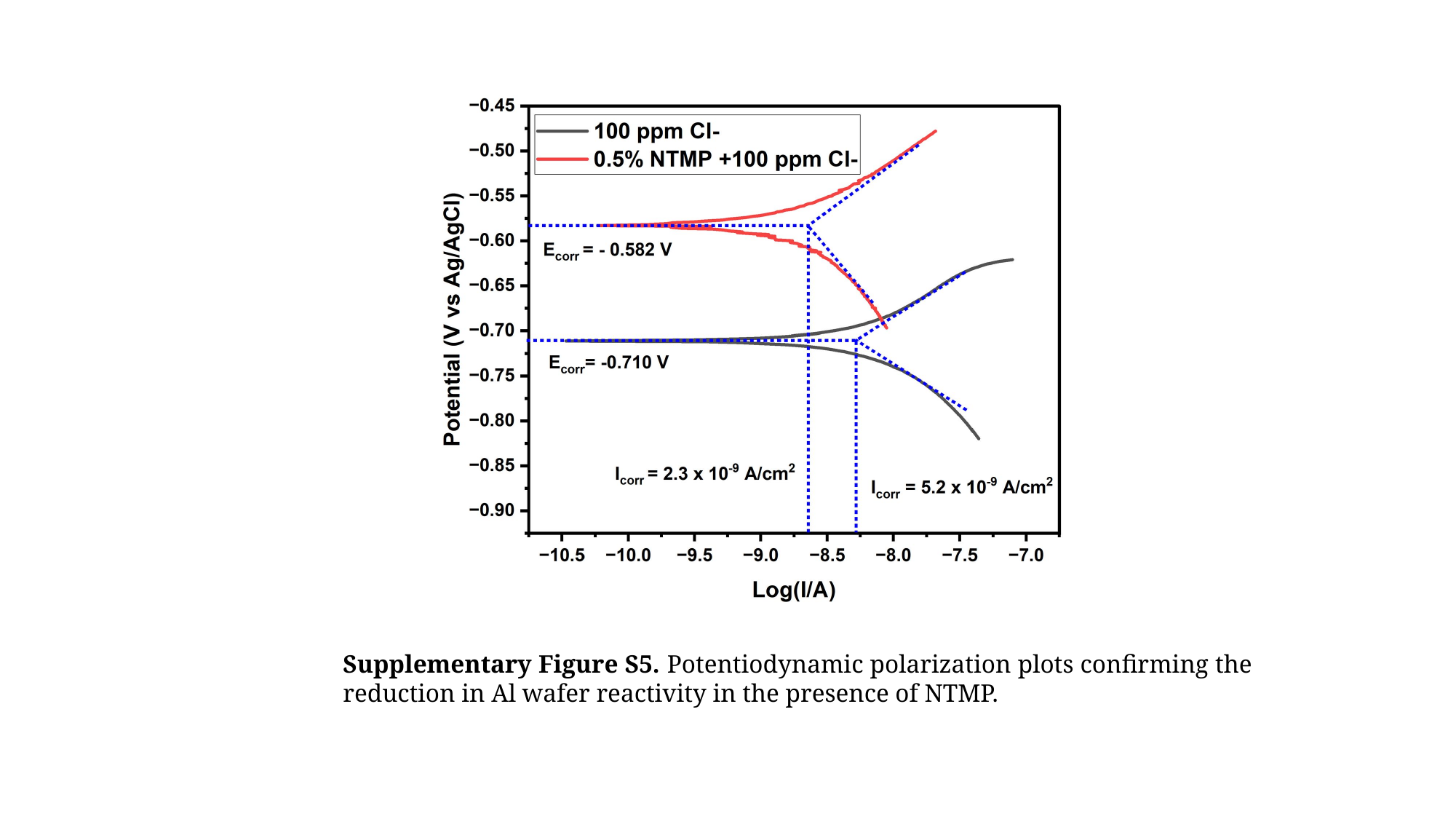

Supplementary Figure S5. Potentiodynamic polarization plots confirming the reduction in Al wafer reactivity in the presence of NTMP.

## Slide 7
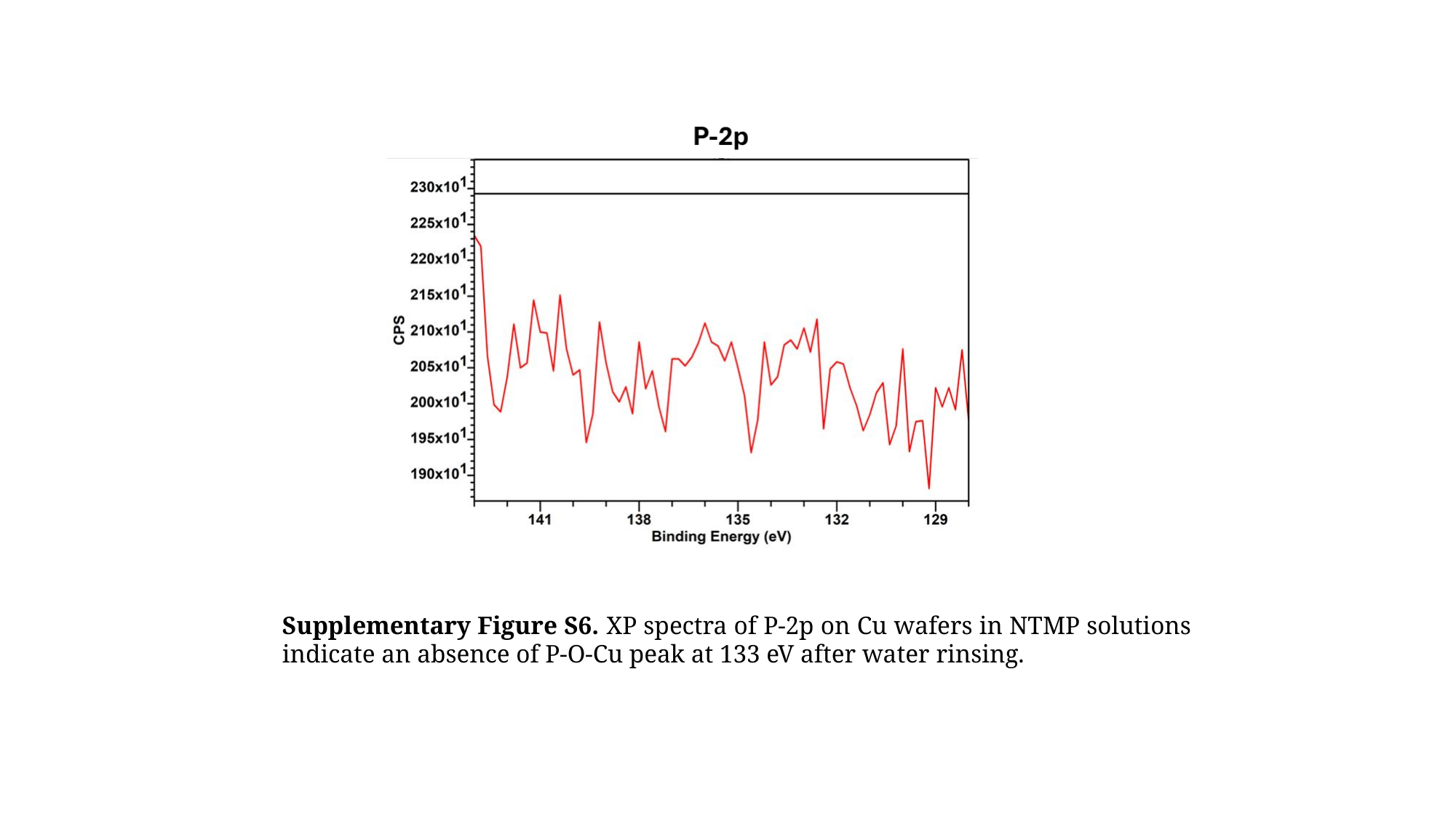

Supplementary Figure S6. XP spectra of P-2p on Cu wafers in NTMP solutions indicate an absence of P-O-Cu peak at 133 eV after water rinsing.
